# Recognition of the D.D.S. Degree by the American Medical Association

**Published:** 1903-07

**Authors:** Eugene S. Talbot


### RECOGNITION OF THE D.D.S. DEGREE BY THE AMERICAN MEDICAL ASSOCIATION.

BY EUGENE S. TALBOT, M.D., D.D.S.

It will be of interest to the dental profession to know that the American Medical Association has recognized the degree of D.D.S. One object of establishment of the Section on Stomatology in the American Medical Association was to try to place dentistry on an equal standard with other specialties in medicine. The members of the Section have labored many years with this idea for a goal. Members of the Association have been the warmest friends from the beginning.

The members of the Section have as their battle-cry, "By their works ye shall know them," and for each meeting a programme has been prepared far above the average dental society programme. Subjects have always been chosen of mutual interest to physicians and dentists to the exclusion of dental technique, since there are many dental societies in which subjects pertaining to dentistry proper are discussed.

When the Section was first organized only those holding the M.D. degree could become members; later, in June, 1887, Dr. N. S. Davis, Dr. W. W. Allport, and myself drew up the following:

"*Resolved*, That the regular graduate of such dental and oral schools and colleges as require of their students a standard of preliminary or general education and a term of professional study equal to the best class of the medical colleges of this country and embrace in their curriculum all the fundamental branches of medicine, differing by substituting practical and clinical instruction in dental and oral medicine and surgery, be recognized as members of the regular profession of medicine and eligible to membership in this Association, on the same conditions and subject to the same regulations as other members."

While this practically recognized the D.D.S. degree, yet the wording of this resolution was so ambiguous that every year the officers of the Section had more or less trouble in admitting members. Thus, at Denver, for some reason unknown to the writer, an edict was issued before the meeting that only those holding the M.D. degree could become members, and some thirty members were lost to the Association. This was unintentional, as it was admitted by the secretary and treasurer after the meeting to have been a mistake.

The Section drifted along under the resolution until 1901, when a new constitution and by-laws were adopted which placed our Section to a greater disadvantage. In the mean time the Section, by the character of its papers and discussions, had placed itself upon an equal standard with other Sections. Nay, more, it has in some respects far superseded the other Sections. The Section on Stomatology has been frequently cited in the past ten years as a model by which other Sections, to be successful, might copy, as witness the following remarks by President Billings at New Orleans: "One of the best conducted Sections of the Association is that on Stomatology. Its efficient secretary has served continuously for sixteen years. This Section is threatened with annihilation since the plan for reorganization was adopted. This should be obviated by the adoption of the by-law proposed last year, which would enable the reputable dentists who have the degree of D.D.S. to become associate members of the Association."

Noticing the predicament and recognizing the high standard of the work of the Section on Stomatology, the Association came to the rescue and passed the following resolution through the House of Delegates:

"DENTAL MEMBERS.

"Dentists who hold the degree of D.D.S. from a reputable dental college and who are members of a recognized local or State dental society may be admitted as dental members on recommendation of the officers of the Section on Stomatology and approval by a majority vote of the Section, the names of such members to be sent to the secretary by the secretary of the Section.

"BY-LAW.

"Dental members shall enjoy the same privileges as regular members and be subject to the same conditions."

It will be seen that not only is the graduate of dentistry placed on an equal standard with the graduate of medicine, but the Association has given the Section on Stomatology great privileges as well. It has given it its own autonomy. This relieves the officers of the general body as well as those of the Section of some of the annoyance which naturally occurred under the old *régime*. The D.D.S. pays his five dollars and receives the weekly journal of the Association, which every practitioner of dentistry or medicine should take. It has been claimed by some dentists that the medical profession has been hostile to dentistry. While it is possible that such may be the case in certain localities and individuals, it is not true of the members of the American Medical Association. When the Section was established at Richmond, Va., in 1881, Drs. Samuel D. Gross, of Philadelphia, Sayre, of New York, N. S. Davis, of Chicago, and Toner, of Washington, were heartily in sympathy with the movement, and took active interest in its welfare from the start. Later, Dr. Marcy, of Boston, not only worked for its interest, but read papers before the Section. It is a well-known fact that these men are all ex-presidents of the Association. The dental profession has no better champions than the present temporary and permanent officers of the Association.

The secretaries from the start have always stood by the Section on Stomatology. Dr. Simmons, the present permanent secretary, has championed our cause throughout the present trouble. He is a warm friend of the Section and always speaks in the highest terms of our work.

I think it safe to say there is not a member in the Association who would not gladly read a paper before our Section upon invitation. We have had many such papers in the past. There has never been the slightest distinction made between the Sections. The Section on Stomatology has as much influence as any other Section.

It has been the aim of the Section to elevate the standard of dental education, and its influence has been felt in universities in the advancement of their years of study, preliminary education, groundwork in medical principles, in the passing of the

Army Medical Bill, and in the establishment of the Army Dental Corps.

Our numbers have not been large as compared with other national bodies. As compared with other Sections in the national body, with the exception of possibly three, we stand very favorably. There is one great advantage, however, in this: when one reads a paper before the Section on Stomatology, upon any subject, he is sure of an appreciative audience. Every person in the room is capable of discussing these papers to the fullest extent.

